# Replication in Energy Markets: Use and Misuse of Chaos Tools

**DOI:** 10.3390/e24050701

**Published:** 2022-05-16

**Authors:** Loretta Mastroeni, Pierluigi Vellucci

**Affiliations:** Department of Economics, University of Roma Tre, 00145 Roma, Italy; pierluigi.vellucci@uniroma3.it

**Keywords:** nonlinear dynamics, chaos, butterfly effect, energy futures, C650, G140, Q470

## Abstract

As pointed out by many researchers, replication plays a key role in the credibility of applied sciences and the confidence in all research findings. With regard, in particular, to energy finance and economics, replication papers are rare, probably because they are hampered by inaccessible data, but their aim is crucial. We consider two ways to avoid misleading results on the ostensible chaoticity of price series. The first one is represented by the proper mathematical definition of chaos and the related theoretical background, while the latter is represented by the hybrid approach that we propose here—i.e., consisting of considering the dynamical system underlying the price time series as a deterministic system with noise. We find that both chaotic and stochastic features coexist in the energy commodity markets, although the misuse of some tests in the established practice in the literature may say otherwise.

## 1. Introduction

As pointed out by many researchers (see, for example, [1]), replication is the key to credibility in applied sciences and confidence in all research findings. With regard in particular to energy finance and economics, replication papers are rare, probably because they are hampered by inaccessible data [1], but their aim is crucial and twofold. First, they wonder if the old results resist if more recent data are added and if the methods are updated, and if not, why this is so. Second, they take into account a large number of recent (or older) articles to check whether the results are still valid when compared with other contributions.

For instance, the same data may be examined by different authors with different methodological approaches. Can the difference in results be explained? Is it possible to distinguish credible results from others that are less so?

Recently, we started to focus on this question by considering, in particular, the findings of the so-called “chaos theory” on the energy commodity markets [2,3,4]. An important reason to be interested in chaotic behavior is that it resembles random behavior (even if they cannot be treated as the same).

In particular, it is interesting to know whether the fluctuations in many time series are really random or they are instead the product of a (complex) deterministic system [3,4,5,6]. The behavior of a completely random system is not predictable anyway. Otherwise, if it were completely deterministic, even if chaotic, its behavior could be predicted in the short term.

It is straightforward that evidence on deterministic chaos would have important implications for regulators and short-term trading strategies, in all financial markets and in particular in energy markets.

Energy commodity prices have been examined over the last 20 years to detect the presence of chaos as an alternative to stochastic models, but they revealed contrasting results: some papers highlighted the presence of chaos, while some others did not, and this has led to a gradual loss of interest in the chaos theory applied to energy commodity markets. For example, the papers we have examined in this field—we have selected only those relating to crude oil, diesel, natural gas and copper—are refs. [7,8,9,10,11,12,13,14,15,16,17], but eight of them fall before 2009 and only three after. (For the discussion of the previous literature, see [2,3,4]).

The conflicting results of identifying chaos in the energy commodity markets can be seen as a replication problem.

Hence, in this paper, we highlight the role of theoretical assumptions of the methods employed in the literature of energy markets. In particular, we show that the mathematical definition of chaos and the theoretical background recalled and discussed here are able to avoid possible errors from misleading results on ostensible chaoticity of the price series.

After showing the importance of the theoretical background in the light of the problem of replication, we also discuss the hybrid approach introduced in [3,4]—i.e., consisting in considering the dynamical system underlying the price time series as a deterministic system with noise—in order to re-evaluate the presence of a chaotic feature in the energy commodity markets. This hybrid approach is based on the introduction of tools that take into account the co-existence of stochastic and chaotic behavior in the same time series, such as modified correlation entropy, noise level estimation and recurrence analysis.

The result is that chaotic characteristics coexist with stochastic ones in the time series of energy commodity prices.

The remainder of this article is structured as follows. Section 2 introduces the chaos definition. Section 3 presents the tools we employ in our analysis, while Section 4 discusses the results. In addition, Section 5 provides the conclusions of our paper.

## 2. The “Core” of Chaos: Its Definition

Who remembers Ian Malcolm, the mathematician of Jurassic Park? In a scene where he tries to explain the chaos theory to Ellie Sattler, he says: “It simply deals with unpredictability in complex systems. The shorthand is the Butterfly Effect. A butterfly can flap its wings in Peking and in Central Park you get rain instead of sunshine.” That is very effective, simple and straightforward.

The chaos definition, however, goes deeper. According to one of the most widely accepted definitions of chaos, introduced by Robert L. Devaney [18] (hence known as *Devaney’s chaos definition*), sensitive dependence on initial conditions, topological transitivity and density of periodic points are the “ingredients” of chaos (for the self-consistency of Devaney’s definition, see the references in [2]). The intuitive meaning of sensitive dependence on initial conditions is straightforward: tiny differences become amplified. It is the most popular property of a chaotic system. Also called “butterfly effect”, it is immediate enough to be cited in a popular film, as we said. This is probably why the “butterfly effect” becomes so predominant that in many contexts, it constitutes, itself, a definition of chaos. There is a lot of numerical evidence for this experimental definition of chaos, but it is not satisfactory, both theoretically and experimentally.

From a theoretical point of view, see, for example, the counterexample 3.3 introduced by Martelli et al. in [19]. Their counterexample shows that, although the “experimental” definition of chaos is easy to check, it defines as chaotic systems those which are not.

As far as the experimental point of view is concerned, however, it has been noted that the time series generated by stochastic systems can also show a sensitive dependence on the initial conditions [20,21,22] and, since chaos theory is an alternative paradigm to the stochastic approach, a problem arises with the definitions—what is chaotic and what is not.

In addition, while some tests for sensitive dependence on initial conditions have been introduced, for the other two properties that build the Devaney chaos definition, we have far fewer tests, and further, no tests for transitivity conditions of the chaos definition have been found [23].

For this reason, it is inappropriate to talk about chaos tests. We should instead refer to the specific property we are going to test. For example, all the papers considered in this article [7,8,9,10,11,12,13,14,15,16,17] resort to the experimental definition of chaos, testing sensitive dependence on initial conditions. However, the implications that the butterfly effect may have in the energy markets make this property interesting to study, as remarked in [2], but… how?

Is there a dichotomy between the butterfly effect and stochastic features? Or is it possible to think of a paradigm that can include both? The answer to this question is, yes, this dichotomy does not need to be a strict rule, as proved in [3,4]. Hence, in the following, we propose a systematic approach to detect the correct tests to work in this “hybrid” framework.

## 3. Methodologies

In this paper, entropy and recurrence analysis tools represent the key methodologies to assess the presence of the butterfly effect. Moreover, we extend some of them in order to deal with the coexistence of chaotic and stochastic behaviors.

In the following, $p_{t}$ and $\kappa_{t}=\ln\frac{p_{t}}{p_{t-1}}$ are, respectively, the price and log returns at time *t*. The time series we will work on is defined as follows: $\left\{\kappa_{t},t=1,2,\ldots ,n\right\}$, n∈N.

### 3.1. Phase Space Reconstruction

Embedding the time series in a phase space is an important research topic on chaotic time series analysis [24]. In this case, the time evolution of returns is represented by the dynamical system that comes out of the phase space independent variables. The asymptotic behavior of the dynamical system is described by an *attractor*, whose dimension provides a measure of the minimum number of independent variables able to describe the dynamical system.

The scalar time series is topologically equivalent to the attractor, which can be reconstructed from a time series by using the method of the time delay coordinate [25,26]. The reconstructed attractor of the original system is given by the vector sequence

(1) $$\zeta \left(i\right)=\left(\kappa_{i},\kappa_{i+\tau },\kappa_{i+2\tau },\ldots ,\kappa_{i+(m-1)\tau }\right)$$

where *m* is the embedding dimension, and τ is an appropriate time delay.

The choice of the time delay τ could be a potential issue. For example, the authors in [27] showed that the chaos measures estimation for stock price data is affected by the wrong choice of τ.

The authors in [8] estimated the optimal time delay as the one where average mutual information reaches its first minimum, obtaining a time lag greater than 1.

In [3,4], we employed the average mutual information (AMI) technique to select a proper value of τ. A proper value of τ can be determined using the first minimum of average mutual information (AMI) function, as done in [8]. The method of false nearest neighbors (FNN), introduced by [28], is an algorithm to estimate the minimal embedding dimension *m*. Let *r* be the threshold on the distance between two neighboring points, k(i) be the index of the time series element for which we have the minimum |ζ(k(i))−ζ(i)|, $\zeta {\left(k\left(i\right)\right)}^{\left(m\right)}$ be the closest neighbor to ζ(i) in *m* dimensions, σ be the standard deviation of the data, and Θ(·) the Heaviside step function, i.e.,

$$\Theta \left(x\right)=\left\{\begin{array}{cc} 0, & x<0,\\ 1, & x\geq 0. \end{array}\right.$$

Hence, the *false nearest neighbor* (FNN) metric is defined as

(2) $$\mathrm{FNN}\left(r\right)=\frac{\sum_{i=1}^{n-m-1}\Theta \left(\frac{{|\zeta \left(i\right)}^{\left(m+1\right)}-\zeta {\left(k\left(i\right)\right)}^{\left(m+1\right)}|}{{|\zeta \left(i\right)}^{\left(m\right)}-\zeta {\left(k\left(i\right)\right)}^{\left(m\right)}|}-r\right)\Theta \left(\frac{\sigma }{r}-\left|\zeta {\left(i\right)}^{\left(m\right)}-\zeta {\left(k\left(i\right)\right)}^{\left(m\right)}\right|\right)}{\sum_{i=1}^{n-m-1}\Theta \left(\frac{\sigma }{r}-\left|\zeta {\left(i\right)}^{\left(m\right)}-\zeta {\left(k\left(i\right)\right)}^{\left(m\right)}\right|\right)},$$

A proper value of *m* can be selected by imposing a threshold ${\mathrm{FNN}}^{*}$ (in our case ${\mathrm{FNN}}^{*}=0.5\%$, as done in [3,4]) so that, if FNN is larger than ${\mathrm{FNN}}^{*}$, the neighbor is false. Since the FNN decreases with the threshold *r*, this is the equivalent of selecting as the embedding dimension the minimum value of *m* such that $\mathrm{FNN}<{\mathrm{FNN}}^{*}$.

### 3.2. Modified Correlation Entropy

Let $\left\{\kappa_{i}\right\}$ be the result of phase space reconstruction described by Equation (1). Hence, the authors in [29] showed that the Kolmogorov–Sinai (KS) entropy can be approximated by the correlation sum

(3) $$C_{m}\left(r\right)=\frac{1}{n(n-1)}\sum_{\overset{i,j=1}{i\neq j}}^{n}\Theta \left(r-∥\zeta \left(i\right)-\zeta \left(j\right)∥\right)\;,$$

where the distance metric is given by the Euclidean norm. From Equation (3), it is possible to achieve an early estimate of the KS entropy

(4) $$K≃\frac{1}{\tau }\ln\frac{C_{m}\left(r\right)}{C_{m+1}\left(r\right)}\;.$$

and its adjusted estimation

(5) $$K≃\frac{1}{\tau }\ln\frac{C_{m}\left(r\right)}{C_{m+1}\left(r\right)}-\frac{D}{2\tau }\ln\frac{m+1}{m}.$$

given by [30], where *D* is the correlation dimension.

Nevertheless, the computation of the correlation sum is affected by noise, which produces errors in these formulas, used instead in the literature so far.

The authors in [31] introduced the *modified correlation entropy* (MCE), which estimates the KS entropy for noisy time series. It is based on the correlation integral derived in [32] and assumes the presence of Gaussian additive noise.

### 3.3. Noise Level

Let $0.1=r_{1}<r_{2}<\ldots <r_{i}<\ldots <r_{L}=0.3$ with a uniform step $\Delta r=r_{i+1}-r_{i}$. The noise level is estimated by means of a linear least-squares method

(6) $${\bar{\sigma }}^{2}=\frac{\sum_{i=2}^{L-2}\left(v_{i+1}-v_{i}\right)\left(u_{i+1}-u_{i}\right)}{2\sum_{i=2}^{L-2}{\left(u_{i+1}-u_{i}\right)}^{2}}\;.$$

as obtained in [33]. It is based on an auxiliary time series $\left(u_{i},v_{i}\right)$, i=1,…,L

(7) $$\begin{array}{cc} u_{i} & =\frac{\left(m-1\right)\Delta r\left(c_{i}-c_{i-1}\right)-r_{i}\left(c_{i-1}-2c_{i}+c_{i+1}\right)-r_{i}{\left(c_{i}-c_{i-1}\right)}^{2}}{r_{i}{\left(\Delta r\right)}^{2}}\\ v_{i} & =r_{i}\frac{c_{i}-c_{i-1}}{\Delta r}, \end{array}$$

where $c_{i}=\ln C_{0}\left(r_{i}\right)$.

### 3.4. Recurrence Analysis

*Recurrence quantification analysis* (RQA) can be considered as another important tool in chaotic time series analysis [34,35]. The *recurrence plot* (RP), introduced by [36], is defined by the matrix

(8) $$M_{ij}=\Theta \left(\epsilon -∥\zeta (i)-\zeta (j)∥\right)\;,$$

where ϵ is a tolerance parameter to be chosen and ζ(i) is derived by Equation (1). Since the distance is symmetric, we have that the matrix *M* is in turn symmetric and, then, the recurrence plot is symmetric with respect to the diagonal, by definition.

The parameter ϵ, which determines the density of RP, can be selected according to the criterion introduced in [37]:

(9) $$\epsilon =k\cdot \max_{i,j}∥\zeta \left(i\right)-\zeta \left(j\right)∥.$$

provided that k<10% [34,38,39].

Related to the RP is the *recurrence rate* [34], which can be defined as follows:

(10) $$RR\left(\tau \right)=\frac{1}{N-\tau }\sum_{i=1}^{N-\tau }M_{ij}.$$

The *recurrence quantification analysis* contains several measures of complexity. Its aim is to go beyond the visual impression yielded by RPs [34].

Some of them resort to the histogram P(l) of diagonal lines of length *l*, i.e.,

$$P\left(l\right)=\sum_{i,j=1}^{N}\left(1-M_{i-1,j-1}\right)\left(1-M_{i+l,j+l}\right)\prod_{k=0}^{l-1}M_{i+k,j+k}\;.$$

As recalled in [34], “processes with uncorrelated or weakly correlated, stochastic or chaotic behaviour cause none or very short diagonals, whereas deterministic processes cause longer diagonals and less single, isolated recurrence points”. From this, it is natural to take

(11) $$DET=\frac{\sum_{l=l_{min}}^{N}lP\left(l\right)}{\sum_{l=1}^{N}lP\left(l\right)}$$

as a measure for *determinism* of the system—percentage of recurrence points which form diagonal structures (of at least length $l_{min}$) over the total number of recurrence points.

Moreover, given the histogram P(v) of vertical lines of length *v*, i.e.,

$$P\left(v\right)=\sum_{i,j=1}^{N}\left(1-M_{i,j}\right)\left(1-M_{i,j+v}\right)\prod_{k=0}^{v-1}M_{i,j+k}\;.$$

it is possible to define the percentage of recurrence points which form vertical structures in the RP, the so-called *laminarity*:

$$LAM=\frac{\sum_{v=v_{min}}^{N}vP\left(v\right)}{\sum_{v=1}^{N}vP\left(v\right)}$$

whereas the average length of vertical structures is given by

$$TT=\frac{\sum_{v=v_{min}}^{N}vP\left(v\right)}{\sum_{v=v_{min}}^{N}P\left(v\right)}$$

and is called the *trapping time*.

## 4. Implications of the New Approach

We now turn to recall the main findings enclosed in [3,4], discussing them in the framework of our approach, i.e., the coexistence of the stochastic and chaotic paradigms.

Before embracing this hybrid paradigm for energy markets, it is very important to determine the two embedding parameters for the reconstruction of the phase space, namely, the time delay τ and the embedding dimension *m*. In Table 1, we recall the embedding parameters of some of the future contracts analyzed in [4], as collected by the U.S. Energy Information Administration (EIA). As we can see, the optimal time lags are not always equal to 1.

**Figure 1:**
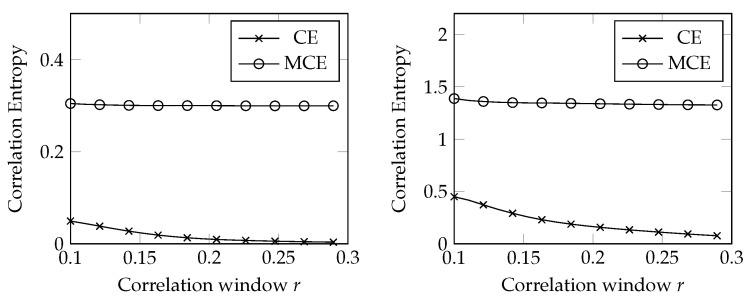
MCE vs. CE; Cushing Crude Oil Contract 1 (on the **left**) and Natural Gas (on the **right**).

According to our framework, the impact of the stochastic component can be initially estimated through the modified correlation entropy. An example of MCE estimation is depicted in Figure 1, where MCE and CE are compared depending on the threshold *r* [4].

In Figure 1, we see the following:

- The KS entropy estimated with a noise-oblivious approach is much smaller than the MCE;
- The CE decays as the size of the correlation window increases, whereas the MCE is rather steady.

Since MCE ≡ CE for noise-free data, these two points show the relevance of the stochastic component in our dataset of prices. The steadiness of MCE is typical of deterministic systems with noise (see Figure 11.3 of [40]).

Connected to this point is the noise level estimation. Few examples of noise level estimation are represented in Table 2 and, as discussed in [4], it shows that the level of noise cannot be ignored.

**Figure 2:**
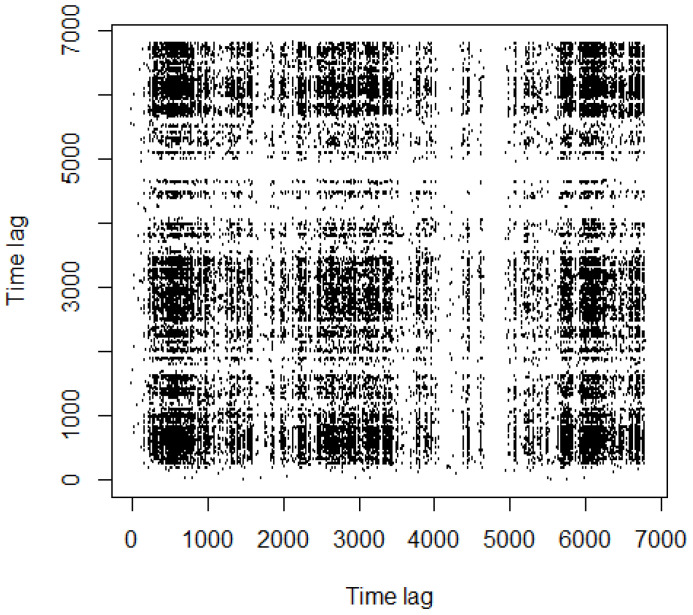
Recurrence plot, copper (6%).

We now turn to prove these insights through the use of recurrence analysis. We show an example of the recurrence plot for copper dataset, examined in [3], in Figure 2, for ϵ=6%.

In Figure 2, black rectangles and single dots alternate along the entire picture. In the recurrence analysis, single points denote noisy behavior [34] because they indicate strongly uncorrelated, fluctuating data, whereas black rectangles characterize *laminar* behaviors. The latter are indicative of states that do not change or change slowly for some time [34,41]. Therein, periods are related to *intermittency*, a behavior of dynamical systems which has been extensively studied in the literature [42,43,44,45].

In economics and finance, intermittency results in the irregular alternation of phases of boom and of depression [46,47].

The authors in [48] showed “how economic intermittency is induced by an attractor merging crisis and how to recognize different recurrent patterns in the intermittent time series of economic cycles by separating them into laminar (weakly chaotic) and bursty (strongly chaotic) phases”. Moreover, intermittency is related to the emergence of bubbles [3,35,49,50].

Intermittency is one of the common routes to chaos [51]. In such a state, the dynamical system switches between two different kinds of behavior called phases. Complex systems which exhibit intermittency can be described by a control parameter *p*. It is characterized by a critical threshold $p_{T}$, which marks the switch from different dynamic regimes [51]. For example, the dynamical system underlying the copper time series is such that $p>p_{T}$, because the laminar phases in Figure 2 are still pretty recognizable ([3]).

White areas or bands in the RPs are caused by abrupt changes and extreme events in the dynamics (*disrupted* typology [36]). They are indicative of transient activities and may reflect an underlying state change [34]. White bands with no recurrent points appear in Figure 2.

**Figure 3:**
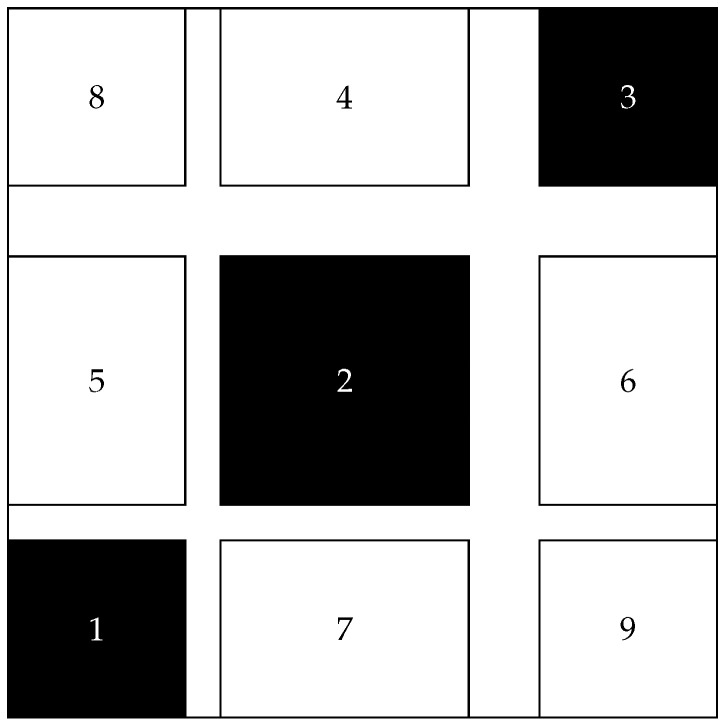
Type I intermittency, positioning of the rectangles in the RP (see Figure 8 in [52]).

Pomeau and Manneville introduced three types of intermittency [42], whose structure were examined in [52] afterwards. According to [52], it is possible to distinguish the kind of intermittency showed by the system by looking at the patterns of RPs. Hence, following [52], the pattern in Figure 2 suggests the presence of a type I intermittency (Figure 3).

**Figure 4:**
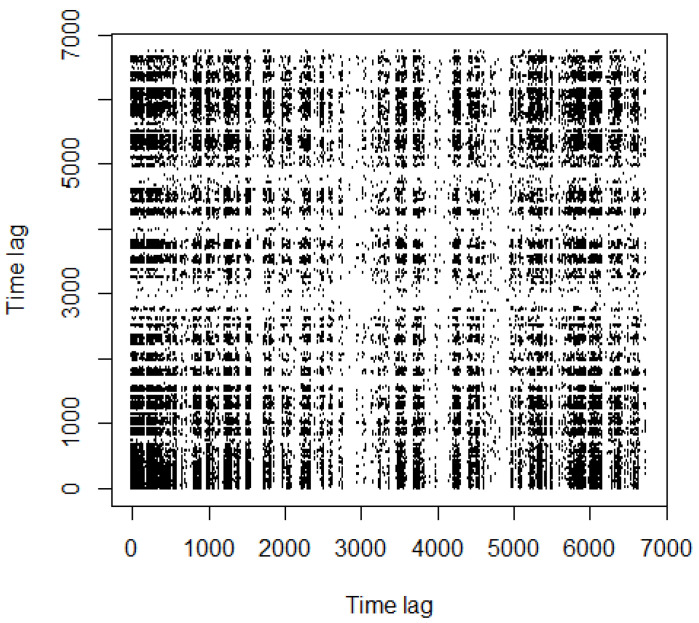
Recurrence plot, natural gas (6%).

Quite different is the RP depicted in Figure 4, for natural gas. We can spot the presence of a larger number of black rectangles, even if they are smaller.

Then it is clear that, in this context, we cannot talk about purely chaotic (or stochastic) time series and that the energy commodity markets follow instead a hybrid paradigm—both chaotic and stochastic. However, do you remember Ian Malcolm’s words? Rearranging them, *the shorthand of chaos is the butterfly effect*. In Section 2, we explained why this cannot be true, and the energy commodity markets give us a *counterexample*. Actually, we estimated the maximal Lyapunov exponent (MLE) for some of the datasets previously examined in [3,4] obtaining: MLE (copper) =−0.78; MLE (oil contract 1) =−0.68; MLE (natural gas) =0.14. From these findings, according to the experimental definition of chaos, we may infer that the natural gas time series is chaotic [2].

MCE, noise level estimation and RP tell us a different story: the stochastic component is too large to be neglected. This result is also confirmed by the measure for determinism enclosed in Equation (11). For natural gas, DET =0.22, which denotes a very high level of stochastic component. The choice of $l_{min}=10$ satisfies the suggestions contained in [34,40]; the choice of ϵ (k=6%) follows the criterion fixed by (9).

## 5. Conclusions

As pointed out by many researchers, replication is the key to credibility in applied sciences and confidence in all research findings. With regard, in particular, to energy finance and economics, replication papers are rare, probably because they are hampered by inaccessible data, but their aim is crucial and twofold. First, they wonder if the old results resist the addition of more recent data and the updating of new methods and, if not, why this is so. Second, they take into account a large number of recent (or older) articles to check whether the results are still valid when compared with other contributions.

While in [3,4] we proved that the contrasting results in chaos theory applied to energy economics are due to replication issues, in this paper, we consider two ways to avoid misleading results on the ostensible chaoticity of price series. The first one is represented by the proper mathematical definition of chaos and the related theoretical background, while the latter is represented by the hybrid approach that we propose here—which consists in considering the dynamical system underlying the price time series as a superposition of deterministic and stochastic systems. This hybrid approach is based on the introduction of tools that take into account the co-existence of stochastic and chaotic behaviors in the same time series, such as modified correlation entropy, noise level estimation and recurrence analysis.

We find that the chaotic and stochastic features coexist in the energy commodity markets, although the misuse of some tests in the established practice in literature—like CE or MLE—may say otherwise.

Our results are in line with the seminal paper by Barnett and Serletis who, more than 20 years ago, conjectured that controversies concerning the application of chaos theory in economics “might stem from the high noise level that exists in most aggregated economic time series and the relatively low sample sizes that are available with economic data” [53]. However, we should observe that the long debate produced by this paper did not answer the question, and, instead, papers dealing with the existence of chaos in economic and financial data continued to be published in the subsequent years [3,4]. Moreover, we do not completely agree with the conclusions enclosed in [53]: “However, it also appears that the controversies are produced by the nature of the tests themselves, rather than by the nature of the hypothesis, since linearity is a very strong null hypothesis, and hence should be easy to reject with any test and any economic or financial time series on which an adequate sample size is available”. We do not believe that “the controversies are produced by the nature of the tests themselves”, and instead we showed here that it would be more correct to speak of the superposition of chaotic and stochastic systems.

The consequences of such findings, though not investigated here, deserve further investigations and suggest, for future works, the adoption of different approaches to predict the behavior of energy commodity prices.

As for future works, artificial intelligence (AI) methods, such as machine learning, offer new possibilities to forecast energy consumption prices. Unlike conventional algorithms, which tend to follow explicit instructions to perform a specific task, machine learning (ML) takes into account various context variables and their mutual relationship while training. For example, in price prediction, supervised learning algorithms can already produce good results, which in turn are applied to time series data. There are already several studies on the predictability of time series data for various applications, including in the energy sector [54,55,56,57].

For the future, it would be therefore good to address these AI/ML-driven techniques for a robust evaluation and estimation of energy consumption prices in the outlook.

## Figures and Tables

**Table 1:** τ and *m* for futures prices (${\mathrm{FNN}}^{*}=0.5\%$).

Futures Contract	Time Delay	Embedding Dimension
Crude oil Contract 1	4	11
Crude oil Contract 3	4	10
Heating oil Contract 1	1	13
Heating oil Contract 3	1	11
Natural gas	1	14

**Table 2:** Noise level estimation.

Commodity Contract	$\bar{\sigma }$	Noise Level %
Crude oil C1	0.02363634	57.9%
Crude oil C3	0.02432642	57.1%
Heating oil C1	0.02032667	51.7%
Heating oil C3	0.02334584	53.5%
Natural gas	0.02591293	40.1%

## Data Availability

The data presented in this study are available on request from the corresponding author.
